# Mapping hospice patients' perception and verbal communication of end-of-life needs: an exploratory mixed methods inquiry

**DOI:** 10.1186/1472-684X-10-1

**Published:** 2011-01-27

**Authors:** Bruce L Arnold

**Affiliations:** 1 Associate Professor of Sociology, University of Calgary, Calgary, T2N 1N4, Canada

## Abstract

**Background:**

Comprehensive "Total Pain" assessments of patients' end-of-life needs are critical for providing improved patient-clinician communication, assessing needs, and offering high quality palliative care. However, patients' needs-based research methodologies and findings remain highly diverse with their lack of consensus preventing optimum needs assessments and care planning. Mixed-methods is an underused yet robust "patient-based" approach for reported lived experiences to map both the incidence and prevalence of *what *patients perceive as important end of life needs.

**Methods:**

Findings often include methodological artifacts and their own selection bias. Moving beyond diverse findings therefore requires revisiting methodological choices. A mixed methods research cross-sectional design is therefore used to reduce limitations inherent in both qualitative and quantitative methodologies. Audio-taped phenomenological "thinking aloud" interviews of a purposive sample of 30 hospice patients are used to identify their vocabulary for communicating perceptions of end-of-life needs. Grounded theory procedures assisted by QSR-NVivo software is then used for discovering domains of needs embedded in the interview narratives. Summary findings are translated into quantified format for presentation and analytical purposes.

**Results:**

Findings from this mixed-methods feasibility study indicate patients' narratives represent 7 core domains of end-of-life needs. These are (1) time, (2) social, (3) physiological, (4) death and dying, (5) safety, (6) spirituality, (7) change & adaptation. The prevalence, rather than just the occurrence, of patients' reported needs provides further insight into their relative importance.

**Conclusion:**

Patients' perceptions of end-of-life needs are multidimensional, often ambiguous and uncertain. Mixed methodology appears to hold considerable promise for unpacking both the occurrence and prevalence of cognitive structures represented by verbal encoding that constitute patients' narratives. Communication is a key currency for delivering optimal palliative care. Therefore understanding the domains of needs that emerge from patient-based vocabularies indicate potential for: (1) developing more comprehensive clinical-patient needs assessment tools; (2) improved patient-clinician communication; and (3) moving toward a theoretical model of human needs that can emerge at the end of life.

## Background

Comprehensive "Total Pain" assessments of hospice patients' end-of-life needs are critical for providing optimal compassionate palliative care. By definition, most hospice patients have an advanced and progressive terminal illness, frequently with multiple co-morbidities, and are approaching the end of their lives. Yet few phenomena are so complex, for both patients and clinicians, and therefore not readily expressed nor easily understood during uncertain and challenging end-of-life transitions. Palliative research into patients' needs, including thematic synonyms including goals of care or quality of care, has employed diverse quantitative and qualitative approaches. Regardless of exemplary research, what constitutes hospice patient needs remains highly diverse with a considerable lack of consensus regarding both domain findings and terminology [1-3]. Because we do not yet have a satisfactory comprehensive understanding of patients' end-of-life needs to guide clinical assessments or planning, barriers to relieving suffering persist. This research draws from social cognitive and mixed-methods frameworks to explore an alternative "patient-based" approach to questions regarding hospice patients' perceptions of needs. Social cognition engages two basic premises that hold considerable promise for this area of research. First, we inherently and ubiquitously impose structures of meaning upon all forms of experience. Second, verbal reports are regarded as indices of cognitive or mental representations of health or illness-related experiences [4]. Mixed methods provide opportunities to capture end-of-life experiences as lived by patients and to convert these phenomena into numeric formats to complement clinical assessment tools. This exploratory research is guided by the following questions: (1) What vocabulary do patients use for expressing their needs? (2) What are the primary needs domains around which verbal expressions gravitate? (3) What is the relative occurrence versus prevalence hierarchies of these domains?

Medical research reporting of background information and methodology often requires brevity. However, the following discussion takes the liberty of breaking with this convention. A blending of quantitative-qualitative and social science-medical paradigms is posited as a way to reframe questions regarding how patient information is being collected and analyzed to better inform patient-clinician interactions. It is therefore reasonable and hopefully helpful to provide readers without specialist knowledge of some of these areas some discussion of select methodological and conceptual assumptions and details.

How hospice patients communicate their perceptions of needs is an essential question less frequently asked but inseparable from questions regarding the content of end-of-life needs. Effective communication is therefore a primary ingredient for understanding and assessing patients' needs as a means for providing optimal palliative care [5-7]. Of particular importance are patients' vocabularies that serve as the currency for expressing their needs as they often differ from clinicians' vocabularies thereby leading to unsatisfying interactions and biased assessments [8,9]. Vocabulary convergence may be confounded because clinicians can unknowingly confuse medical words and expressions with those of everyday language used by patients. For example, differences between clinician and patient vocabularies have been reported to limit patients' ability to express their complete lists of concerns to less than 50% of their actual needs [10]. Recently, indirect modes of communication through metaphors and analogies has not only been shown to improve patient-clinician communication but their exclusion may actually cause forms of patient harm [11]. Identifying patient-based specific vocabularies through their narratives therefore hold considerable promise for enabling clinicians to more precisely identify and assess the patients' need domains [12].

It can be tempting to embrace patients' vocabulary as constituting empirically satisfying end-of-life narratives because they allegedly permit ready access for identifying need domains. This would be folly as it is likely to produce an incomplete reading of what is being reported. Rather than assuming verbal reports can be taken at face value it is prudent to first consider their end-of-life context; and second, how domain importance is ranked by patients. First, verbal reports represent indices of elaborate cognitive processes through which we have the capacity to attribute meaning to our experiences. We tacitly use a series of elaborate cognitive sampling schema to select and encode various perceptions of experiential stimuli into appropriate words for communication [13,14]. However, this is often problematic for hospice patients. End-of-life experiences can be ambiguous, unfamiliar, threatening, and stochastic thereby rendering previously reliable cognitive and language categories impotent [15,16]. Hospice patients' expressions of experiences are therefore verbally encoded manifestations of emerging meanings and properties which unfold during the narrative. Some emergent properties may be amenable to familiar and coherent verbal encoding but the more incongruous experiences [17-19] will take symbolic forms using subtle linguistic nuances often outside intentional awareness. Second, domain needs are typically identified and ranked by their occurrence by counting patients who report a particular need. This can be misleading. Instead, a more discriminating view of how patient's prioritize the relative importance of needs is available using the prevalence of verbal utterances associated with each specific need. Patients' reports of needs cannot be taken as prima facie but require specific methodological choices that attend to their multifaceted phenomenology.

## Methods

This research used a mixed methods conversion design to collect qualitative narrative data which was subsequently converted into and analyzed in quantitative-numeric form [20]. Mixed methods are not intended to replace, but instead draw from qualitative and quantitative strengths while minimizing their limitations to offer a more pragmatic and pluralistic mode of inquiry [21-23], including end-of-life research [24]. The mixed methods design used here employs a qualitative study of patients' lived end-of-life experiences to inform the development of a summary quantitative hierarchal measure of their perception of needs without ignoring either methodological tradition. Although the phenomenon of end-of-life experience of needs appears across qualitative and quantitative paradigms in this research the distinction between "lived experience" and "measure" reconciles the phenomenon to its respective method and paradigm [25].

This feasibility study was restricted to a three month period and therefore 30 hospice patients for pragmatic reasons. This author's visit to the Institute for Palliative Medicine at San Diego Hospice research site to conduct interviews and test the feasibility of the research design was limited to this time frame. Hospice patients are a fragile and dynamic study population subject to high levels of attrition that presents specific challenges to participant selection criteria and sampling strategies. The inclusion criteria for eligible patients were: (1) currently receiving inpatient or homecare through San Diego Hospice; (2) adult English speaking; (3) ability to sustain wakefulness, attention, and effort for approximately 20 minutes or longer; (4) provision of documented informed consent; and (5) no significant cognitive impairment or minimally cognitive impaired (MCI) but at least oriented times 3 with no documented altered mental status (confusion, disorientation, delirium, psychosis). MCI may slightly alter memory, language, or judgment but these are not severe enough to interfere with patients' day-to-day life, usual activities, or their ability to provide informed consent. Exclusion criteria included the large proportion of patients receiving hospice care in ancillary facilities; such as, skilled nursing (SNF) or residential care (RCFE) facilities. The research design and protocol was approved by the Conjoint Faculties Research Ethics Board of the University of Calgary and the Institutional Review Board of the Institute for Palliative Medicine at San Diego.

During the three month period, the 493 hospice patients who were not eliminated by the exclusion criteria were assessed for eligibility by an experienced research nurse. On occasion consults with patient's palliative physician and other clinical team members were undertaken to clarify eligibility status. Each of the 181 patients deemed eligible were contacted by telephone by the research nurse, informed of the study, informed of the role of the principal investigator who conducts the interviews, and invited to participate. Some patients declined to participate because they were not interested, were experiencing discomfort, or were busy with visitors or medical appointments. The principal investigator, who is not a member of patients' care team, also called each of the patients who expressed interest to provide more project detail, answer questions, and establish a convenient time to visit for the interview. Some patients agreed to participate but died prior to an interview. This selection process produced a reasonable feasibility sample of 30 patients who generously participated in the interview.

Table 1 introduces the reader to the available descriptive demographic and illness characteristics of the patients who participated in this study. This sample was not intentionally equally stratified by gender (50:50) but occurred naturally. Most of the participants were married (43.3%) and predominantly white non-Hispanic (66.7%). With the exception of Jewish patients (6.7%), patients tended to be almost equally distributed across Catholic (20%), other Christian denominations (26.7%), non-denominational but spiritual (26.7%) or having no religious or spiritual beliefs or affiliations (20%). Most patients' primary diagnosis when entering into hospice service was some form cancer (60%). A smaller proportion of patients' primary diagnosis was a form of lung (10%) or cardiovascular disease (10%). The "other" patients (20%) were diagnosed with various diseases including neurological, renal failure, and failure to thrive. Most patients were limited by endurance considerations (73.3%), some by incontinence (16.7%), and a few by loss of hearing (6.7%). Patients' functional limitations were carefully taken into account during interviews.

**Table 1:** Patient Sample Characteristics (N = 30)

Age		Mean 72.2	SD 13.1
		**N**	**%**

Gender	Male	15	50

	Female	15	50

Marital Status	Married	13	43

	Single	4	13

	Widowed	4	13

	Divorced	5	17

	Unknown	4	13

Race-ethnicity	Unknown-other	3	10

	White non-Hispanic	20	66.7

	Black non-Hispanic	2	6.7

	Asian	2	6.7

	White Hispanic	3	10

Spiritual Identity	Catholic	6	20

	Jewish	2	6.7

	Christian	8	26.7

	Other Spiritual	8	26.7

	No Affiliation	6	20

Primary Diagnosis	Cancer	18	60

	Lung	3	10

	Cardiovascular	3	10

	Other	6	20

Functional Limitation	Endurance	22	73.3

	Incontinence	5	16.7

	Hearing	2	6.7

	Other	1	3.3

Phenomenological-based interviews were used to unobtrusively collect patients' narratives of their lived experiences and perceptions of end-of-life needs. Interview duration was determined by the patient and ranged from 15 minutes to 1 ½ hours. Patient interviews were digitally recorded and transcribed verbatim to produce end-of-life narrative data. This form of interview is designed to minimize selection bias introduced by researchers, predetermined questions or lines of questioning [26-29]. To further reduce priming patients' responses only one introductory question was used: "We are continually trying new ways to learn how to take better care of our patients. Now that you are at the end stages of your life, you are having a lot of important experiences many of which we don't know much about and would like to learn from. Could you please tell me about them as best as you can in your own words?" This allows patients to "think aloud" to formulate authentic descriptions of experiences into narrative data during which the researcher is engaged in compassionate listening [30]. Considerable attention was given to conduct the interviews in a friendly, supportive, and non-hurried pace, especially for patients who require time to reflect and recall events, places, and people. Moments of varying durations of silence occur because patients often do not have a familiar script to follow for expressing their experiences and needs. Patients were informed that these normally occur, are useful and important, and were invited to take their time during these moments. However, patients occasionally appeared to become stuck in extended silence, even become uncomfortable, or ask for the interviewer for direction. Repetition questions by the interviewer provided an appropriate strategy to segue the patient from their most recent comment through an awkward silence. For instance, during an awkward silence the patient would be asked; "You mentioned something about ...." When patients' comments were confusing or paradoxical, the interview would paraphrase the last comment, "do you mean....." or "so you are saying ......" Narrative topics are not randomly chosen by patients from a plethora of past, current, and future phenomena. Instead, topics emerge through cognitive selection processes representing explicit and implicit prioritized needs and goals [31]. However, unlike most narratives, patients' "stories" do not necessarily follow a logical sequential beginning, middle, and conclusion script. Nonetheless, narratives produce "thick descriptions" essential for configuring their prioritized interpretation of events, historical sequences and associations, and meaning that make up the significance of lived-experience for participants [32,33].

These complex narrative data require systematic investigation for identifying the explicit and tacit cognitive dimensions of needs. At first glance, it appears that hospice patients are embedded in a forest of familiar and unfamiliar experiences with precise and satisfying verbal expressions therefore inherently remaining somewhat elusive. However, verbal utterances of experiences are not random but represent valid intra-personal cognitive sampling processes [34]. And, ample evidence supports verbal encoding as a relatively valid measure of cognitive structures and processes, especially if persons "think aloud" rather than react within the confines of research questions [35-37]. So although narratives represent emerging unobservable cognitive phenomena we are not left with arbitrary subjectivity but with layers of structured phenomena [38,39].

Contemporary Grounded Theory methodology (GTM) provides coherent, flexible, and pragmatic inductive-deductive procedures well suited for analyzing data whose structures are multidimensional and unfamiliar [40,41].These procedures facilitate a balance of remaining sensitive to participant's lived experience with judicious use of previous research to go beyond the empirical to generate potential explanatory conceptual frameworks. These types of data present significant project management and analytical challenges. Advanced qualitative analytic software was therefore used as a resource to facilitate GTM. QSR-NVivo8 is a qualitative analytic tool well-suited for this purpose and guided by systematic and pragmatic scientific-based coding using grounded theory procedures to discover manifest and underlying structures in qualitative data [42-45]. NVivo does not replace careful and systematic decisions required by researchers. Instead, it is designed to enhance internal validity through managing axial coding processes to contribute towards credibility, authenticity, confirmability, and dependability [46]. It was not possible to revisit patients to clearly establish the credibility of their comments. However, phenomenological interviews were chosen as the data collection instrument because they enhance authenticity and credibility. Beginning with the listing of patient-specific vocabulary and consulting with team colleagues when opaque coding issues arose lends to the dependability or inter-rater reliability of the analysis. This researcher continually monitored the possibility of introducing personal selection bias into the analysis and carefully utilized GTM procedures to promote confirmability. In addition, ongoing considerations regarding negative-coding and types of theoretical validity (Types 1 and 2) contributed to reducing selection bias errors of inclusion and exclusion. However, these types of software are not silver bullets and their utility is diminished if the research does not pay close attention to her or his inductive and deductive decision-making throughout the analysis [47,48]. Albeit brief, the discussion of the analytic process below introduces some features regarding the synthesis of qualitative and quantitative data analysis.

GTM involves a constant comparison method of coding and analyzing data through three basic iterative coding stages: open or initial coding, axial coding, and selective coding [49]. All approaches to research require careful attention to decision-making to reduce bias and error. Because of the emphasis placed upon the researcher-data interaction dynamics qualitative research calls for more rigorous efforts to recognize preconceptions and bias [50]. However, GTM decision-making is heuristically guided by acknowledge tentative ideas or concepts. The analysis of these interview data was initially influenced by Dame Cicely Saunder's concept of "Total Pain" [51]. As the analysis developed, the unexpected frequency of positive experiences reported by hospice patients led to a cursory inclusion of some of the humanistic psychological and palliative care literature. In this research, the open coding process began with a simple frequency listing of the vocabulary used by patients. This led to preliminary groups of terminology, patient-based synonyms, and syntax tentatively related to patients' needs was examined in their sentence and paragraph contexts. Similarities and difference were coded into nonhierarchical NVivo free nodes as independent concepts. Extensive iterations of NVivo search queries produced more concepts thereby increasing the number of nodes. The axial coding stage again employed similarity-contrasting analytic criteria for specifying potential relationships, commonly referred in GTM as "theoretical sampling". The growing numbers of codes were organized into (tree-branch) nodes with more than one dimension. Selective coding further specified the emerging characteristics of these multiple dimensional concepts (nodes) resulting in the emergence of the seven core patient-based perceptions of end-of-life needs. For example, social needs included family members and friendships, and physiological needs included pain issues, treatment comfort concerns, medication, and disease specifics. NVivo provides options that facilitate assigning numeric values to nodes. This allowed the conversion of core nodes into matrices that were downloaded into an Excel spreadsheet to display hierarchical structures of needs that have emerged through the GTM analysis of patients' narrative data. The results do not exactly match the neither "Total Pain" categories nor human needs posited by humanistic psychologists, but their similarities are worthy of consideration.

## Results

**Figure 1:**
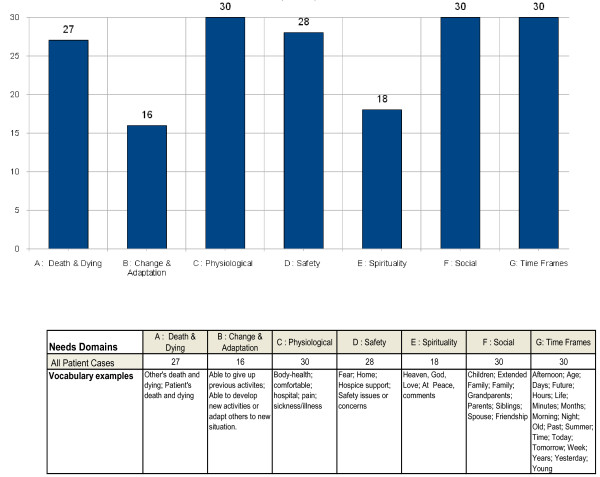
**Occurance of hospice patient reported perception of needs domain (n = 30)**.

**Figure 2:**
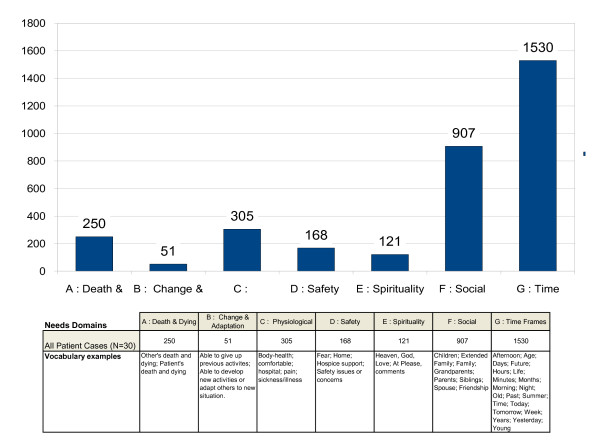
**Prevalence of Hospice Patient Reported Perception of Needs**.

Figures 1 and 2 present the quantified results of the narrative analysis of 30 hospice patient phenomenological interviews. The NVivo analysis uses patient-based vocabularies for uncovering predominant domains around which their verbal expressions of needs experiences tend to gravitate in both their occurrence (Figure 1) and prevalence (Figure 2).

Patient needs research typically inquires into the number of patients' who explicitly state various end-of-life needs. Figure 1 extends this approach by including both explicit and implicit expressions of hospice patients' needs. For instance, patient's comments about death and dying appear to be structured with direct reference to themselves but also through indirect associations with others who they have known and died. Using past experiences to make sense of the present is expected. The use of time-related categories among all patients suggests a symbolic reference for sequentially organizing past, current, and anticipation of possible future events. All patients refer to social relationships at the end-of-life, and as is expected, thoughts regarding their medical-health related needs. Most (N = 28) patients appear to have thoughts regarding their safety. For instance, they comment directly on their hospice support needs but also symbolically through associations regarding security with their home. Only about half of the patients directly address needs related to end-of-life change and adaption (N = 16), and self-actualization or spiritual experiences (N = 18).

Figure 2 shifts the focus from how many patients' report various needs domains to how frequently these needs are expressed by all 30 patients. Compared with the occurrence of a statement regarding need, prevalence offers a more robust indicator of the strength or relative importance assigned to each domain. Direct references to their or others death or dying (N = 250) are significantly over shadowed by symbolic time-related comments expressing and spatially locating present experiences within their life course (N = 1,530). Patients' frequent (N = 907) references to various people in their lives speak to the importance placed upon needs associated with social relationships. Comments regarding physiological needs are ranked only as a third priority (N = 305) and safety needs (N = 168) fourth suggesting either these basic needs are already met or others have become more focal at this point in their lives. While patients do not frequently introduce comments related to unfolding change and adaptation (N = 51) or self-actualization needs (N = 121) the fact that these topics do emerge unprompted speaks to their importance and potential as end-of-life needs among a subpopulation of patients.

## Discussion

Being able to identify both the occurrence and prevalence of patient-based perceptions of end-of-life needs is a fundamental ingredient for offering optimal palliative care. The mixed-method research design provides improved patient-based access to both rather than adopting differential qualitative-quantitative paradigms which generate exemplary studies but may also be prone to partitioning the field of inquiry. The thrust of this research is to explore patient-based perceptions of end-of-life experiences in such a way that allows discovery of unknown dimensions of needs, some of which are not easily expressed or identifiable [52].

Findings illustrate that when provided the opportunity hospice patients' are independently able to make-sense of familiar, unfamiliar, and uncertain end-of-life experiences through their native vocabulary and intuitively organize them around needs domains. Patients employ their own vocabulary to explicitly and implicitly report these lived experiences in the absence of guiding research questions. Some of these expressions can be easily misunderstood or overlooked given their manner of verbal expression which may appear as casual comments unassociated with clinical vocabularies or patient needs. Insights into which verbal utterance patients' assign to needs oriented perceptions and how frequently they report them have direct and significant implications for understanding and assessing their relative importance. Patients' narratives suggest the following needs domains in terms of relative importance: time, social, physiological, death and dying, safety, spirituality, change & adaptation.

Previous inquiries into end-of-life needs have included exemplary original data collection and extensive literature reviews. Some place focus solely upon patients while others have included patients, families or care givers. Researchers also use diverse methodologies and terminologies for needs-related phenomena. The research presented here reframes conceptual and empirical questions; direct comparison with previous undertakings therefore tends to be awkward and requires one to proceed cautiously. This research is only in the context of previous patient-focused findings in order to limit this comparative handicap. Overall, this research both supports and extends what is currently known about hospice patients' perceptions of needs domains. For ease of comparison, findings from this research are sequentially organized into four groups: physical and social, psychological, spiritual, and time domains.

Physical and social needs domain findings in this project are not unexpected as they mirror ubiquitous and well documented patient concerns regarding pain and symptom management, social support, and other relationship-oriented concerns [53]. In contrast, when comparing the prevalence of comments it appears hospice patients place considerable more emphasis upon social rather than physiological needs. The considerable importance of social relationships supports "belongingness" as a primary human need and one whose significance can increase near death. We might infer that less emphasis placed upon physiological needs may be the result of quality pain and symptom management by hospice staff.

Psychological phenomena are heterogenic, complex, and therefore employ diverse terminology. Again, this limits direct comparison between research findings, especially if the taxonomies vary between researcher and patient vocabularies. Regardless, patients' types of psychological needs are usually reported as wanting more control, quality of life, and an overall sense of well-being [55,56]. These psychological categories are analogous to our patients' comments included in Change & Adaptation and Safety domains with the later being reported by more patients than the former. Their occurrence reflects previous research findings. Unexpectedly their prevalence suggests they may be less important for patients than physiological and especially social needs. Other studies report patients' psychological concerns can be met through social interaction; for instance, patients say they need to talk with others who listen compassionately about their dying [57]. This suggests psychological needs may be tacitly embedded in comments regarding relationships in the social domain.

Spirituality lies at the frontier of palliative care. It is now recognized as a salient end-of-life need offering to reduce overall suffering and remarkable existential possibilities to patients [58]. However, the relatively low prevalence of patient comments representing spirituality lends tenuous support to previous claims of spiritual well-being or relationships with God as a core need among the dying. But spirituality can be challenging to capture as it appears in many forms that often extend beyond the boundaries of being readily recognizable or accessible to conventional vocabulary, and intentional thought. The research findings here, for example, include the under-researched "ignored" transcendent qualities of hospice patients' spiritual experiences that lie on the horizon of our current understandings [59,60]. Unprompted, patients shared stories of unexpected ontological and identify-shifting experiences that appear to consist of altered states of being. As one patient informed me, the phenomenology of these experience are difficult to express directly, "It's almost frustrating because you can't articulate it, and I want to so much because it is very possible for everyone to have what I have!" Patients reporting these transformative experiences move beyond our conventional views of future oriented desires or goals. Instead, they appear to undergo a type of awakening gestalt: just being and letting go of attachments to conventional desires [61,62]. We might therefore infer that this domain of experience is better considered not as a need but as a post-need phenomenon or patient self-realization.

The prevalence of patients' comments associated with time is perhaps the most intriguing discovery. Objective measures of time, such as days, weeks, and years emerge throughout their narratives. The phenomenon of time is understudied but nonetheless ubiquitous in palliative care. Patients' basic sense of time is one of the fundamental perceptual building blocks of their worlds destroyed during traumatic illness events [63]. Adverse ontological sequelae include patients' disrupted conceptions of self and states of existential chaos. However, our innate need to impose structures of meaning generates a cognitive adaptive reconfiguration of these events [64]. Past, present, and future events are not just temporal or defined in terms of their content but are relational and require restructuring in response to awareness that our life span time is also both uncertain and finite. Patients' systematic reference to time represents the process through which they tacitly attempt to assert a degree of ontological security; a renewed semblance of order, identity flux, and connection to life events.

## Conclusions

This exploratory study has a number of limitations that should be considered. First, generalizability of findings would be premature given the size of the sample and that patients were drawn from one palliative organization. Sample size also prevents analysis of subpopulations, such as the specific needs of persons with particular diseases. Second, since data collection and analysis was undertaken by one person, researcher selection bias is a potential limitation regardless of previously mentioned safeguards. However, the strength of this study was the mixed-methods design which illustrates a viable way to bridge qualitative-quantitative research distinctions to reinvigorate our search for more comprehensive and "bottom-up" understanding of patients' lived experiences of total pain. This study offers preliminary patient-based evidence; patients' use their own vocabulary to directly and tacitly communicate their end-of-life domain needs through narratives. Findings from this research also offer preliminary scaffolding for moving beyond description of diverse hospice patient populations. They serve as a compass for two possibilities for reducing total pain among palliative patients. First, they provide the direction for developing sensitive clinical communication-assessment tools using patient-based vocabularies. There are few if any familiar conversational scripts for terminally ill persons to clearly express what they are experiencing and what their needs or concerns are. This leaves patients frustrated and clinicians often unclear exactly what is occurring with their patients thereby limiting the delivery of optimal palliative care. For example, knowing how patients' use everyday language to try to represent their multidimensional end-of-life needs has direct clinical assessment and therefore palliative care goal implications. Second, the findings suggest a promising starting point for a palliative care-specific theoretical model to generate a deeper and more holistic understanding and innovative responses to end-of-life needs. For example, which needs are relatively constant and which covariates impact the specific needs of hospice patient sub-populations. Explanation begets prediction and therefore increased quality of care. Such a model is sadly missing in the palliative care resource tool-box. However, placing increased emphasis upon the prevalence of patients' perceptions provides a revised ranking of their needs reminiscent of Maslow's Hierarchy of Needs. Recently Maslow's Hierarchy of Needs has been touted as a possible theoretical framework with clinical applications for more comprehensive and compassionate palliative care [65-67]. Maslow advocated a multidimensional theory of needs driven by our innate search for meaning. Often overlooked, he posited needs as having both conscious and unconscious characteristics with emphasis upon the latter, the emergence of new and yet undiscovered needs, and nonlinear variance between the relative importance of needs satisfaction in diverse contexts [68]. There appears an encouraging juxtaposition between Maslow's model and the results of this research are worth further consideration. It is timely for the next chapter in the palliative care paradigm shift: to more fully integrate and implement patient-based multidisciplinary teams using mixed methodologies for advancing compassionate care.

## Competing interests

The authors declare that they have no competing interests.

## Authors' contributions

The ideas, design, data collection, analysis, conclusions, and research limitations presented here are the sole responsibility of the author.

## Pre-publication history

The pre-publication history for this paper can be accessed here:

http://www.biomedcentral.com/1472-684X/10/1/prepub
